# New Appliances and Things Medical

**Published:** 1894-06-16

**Authors:** 


### New Appliances and Things Medical.

[All preparations, appliances, novelties, &c., of which a notice is desired, should be sent for the Editor, to care of The Manager, 428, Strand, London, W.C.]

### PIPERAZINE AND PHENOCOLL IN THE TREATMENT OF GOUT.

The production of piperazine marks an epoch in the history of synthetic medicinal compounds, since it is the first organic base, the product of the laboratory, which has been employed to expedite the removal of uric acid from the organism. The researches of Sir William Roberts on uric acid and its compounds clearly show that the deposition of urates in the tissues, or in urine after standing, is not the simple process that was at one time supposed, and that the laboratory experiments on the solubility of urates by alkalis and other substances has no exact counterpart in the tissues of the organism. In spite of the fact that the neutral urates of lithium, sodium, &c., which are formed in the presence of excess of alkali, are comparatively soluble compounds, yet as a matter of fact, the administration of large doses of these carbonates or other organic salts of lithium or sodium are comparatively ineffectual in the removal of excess of uric acid from the animal system. Indeed, Sir W. Roberts suggests a free flushing out of the system by a greatly increased diuresis as a more rational method than the free administration of alkalis. Laboratory experiments show that piperazine is seven times as powerful a solvent of uric acid as is the carbonate of lithium, but from what has been said above we must not jump to the conclusion that it is necessarily seven times as expeditious in the removal of uric acid from the tissues. Nevertheless, clinical experience goes far to show that piperazine does measurably and consistently increase the output of uric acid, and that renal calculi, gravel, and gouty deposits eventually become disintegrated and finally removed from the body, when the treatment with piperazine is perseveringly adhered to. In our own hospital experience, in which we gave piperazine a somewhat extensive trial, we cannot say that we met with such success in the treatment of acute cases of gout, and those in which deposits had already occurred, as we did when we employed it as a prophylactic in chronic cases against exacerbations of the symptoms. We believe that it is a more valuable drug when employed as the daily scavenger of uric acid from the blood and tissue juices, than when it is employed to remove large depositions, either in the form of tophi, renal calculi, or gravel. On the other hand, its employment by German physicians in these latter cases appears to have met with considerable success. Piperazine has a formula which may be represented as—

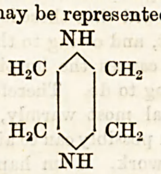

and its stability as a chemical unit is, no doubt, largely due to its symmetrical molecular structure. In combination with uric acid it is soluble at the temperature of the body in 40 parts of water. The statement that it causes albuminaria appears to be founded on the observation that the urines of patients taking piperazine have given a precipitate with picric acid; since piperazine itself gives a white precipitate with this reagent, further proof is necessary. Probably the best method of administering this drug is to give it in 10-grain doses in half-a-pint of ærated water three times during the day. Phenocoll is closely related to phenacetine; in fact, it is this latter body in which one $(NH)_2$ has been substituted for one atom of H, a substitution which enables it to unite with acids to form salts. Its ready solubility in water gives it a distinct advantage over phenacetine, the therapeutic properties of which it otherwise closely resembles. When given in combinatian with piperazine in cases of gout complicated by neuralgic pain, sciatica, or the so-called uric acid headache of Haig, there seems to be a general consensus of opinion as to its efficacy.

[We shall be glad to receive any account of the experience of English physicians in the use of the above drugs. The literature in English periodicals as regards these drugs is at present so meagre that we had had to depend for our data almost entirely on foreign sources.—ED.]

